# Community intervention programs for sex offenders: A systematic review

**DOI:** 10.3389/fpsyt.2022.949899

**Published:** 2022-11-24

**Authors:** Sofia Barros, Cláudia Oliveira, Eduardo Araújo, Diana Moreira, Fernando Almeida, Anita Santos

**Affiliations:** ^1^Department of Social Sciences and Behavior, University of Maia, Maia, Portugal; ^2^Center for Psychology, University of Porto, Porto, Portugal; ^3^Centre for Philosophical and Humanistic Studies, Faculty of Philosophy and Social Sciences, Universidade Católica Portuguesa, Lisbon, Portugal; ^4^Laboratory of Neuropsychophysiology, University of Porto, Porto, Portugal; ^5^Institute of Psychology and Neuropsychology of Porto–IPNP Health, Porto, Portugal; ^6^Centro de Solidariedade de Braga/Projecto Homem, Braga, Portugal; ^7^Hospital Lusíadas Porto, Porto, Portugal; ^8^Institute for Research and Innovation in Health, University of Porto–i3S, Porto, Portugal; ^9^Institute of Biomedical Sciences Abel Salazar–ICBAS, Porto, Portugal

**Keywords:** offender, sexual violence, community program, reintegration, risk, recidivism

## Abstract

Sexual violence is a phenomenon that negatively impacts the victims' physical and psychological health and well-being. Sex offenders tend not to take responsibility for their actions, have difficulties in emotion regulation and impulse control, paraphilias or other disorders, so they are a difficult group to treat. In addition, the available psychological treatment programs tend to have inconsistent and, sometimes, undesirable results. This systematic review aimed to analyse the recidivism rates of sex offenders treated in community settings. According to the PRISMA guidelines, a systematic search in three databases, EBSCOhost, PubMed, and Web of Science, and a manual search was performed. A total of 319 empirical studies using quantitative methodologies were identified, 27 of which were selected for full-text analysis. In the end, 15 studies were included, published between 1996 and 2020. The objectives, intervention approach, instruments used, and the main results and conclusions were extracted from each study. The studies explored different types of sex offenders, such as: violent sex offenders (e.g., rapists), child abusers, and child abusers with pedophilia (and/or other paraphilias). Results showed that most of the programs had a cognitive-behavioral approach (*n* = 13). Overall, the interventions appear to be effective in reducing recidivism rates, and some of them led to improvements in other outcomes, such as cognitive distortions, accepting responsibility, victim awareness and empathy, emotional regulation, and offense supportive attitudes. Limitations and implications for future studies were discussed.

## Introduction

Sexual offense has acquired increasing visibility, constituting a major source of concern and social instability (1). This is considered one of the most serious forms of violence that occurs in Western societies, due to its impact on both physical and psychological health of the victims and their families (2).

According to the World Health Organization (3), sexual offense can be defined as any act of a sexual nature, in a consummated or attempted form, usually of a coercive nature, which may involve physical contact and the use of violence. According to Bonnar-Kidd (4), it can include a wide range of behaviors, such as anal, vaginal, and/or oral penetration, urinating in public, caressing, human trafficking, or unwanted comments. Mostly, sexual offenses are perpetrated by men, of any age, against women or children. However, they do not depend on the existence of any relationship between the victim and the offender, and may occur in different contexts (e.g., home, work), including in public spaces (e.g., gardens) (5).

Although there have been developed typologies that account for deviant sexual behavior, this classification has proven to be problematic (6). Sexual offenders exhibit diverse and heterogeneous characteristics, such as gender, age and context of deviant behavior (type of paraphilia, type of victims, psychiatric comorbidities, and the association with addictive behaviors). This results in different types of crimes and illegal sexual behaviors, requiring individualized treatment planning that considers the specific risk factors and criminogenic needs (e.g., emotional regulation deficits, social difficulties, offending supportive beliefs, and deviant arousal) [e.g., (7)]. This heterogeneity seems to have been challenging effective risk management and treatment of sex offenders (6), remaining controversy over whether or not sex offenders can be effectively treated (8).

There have been some traditional typologies for sex offenders, such as: adult rapists or violent offenders; child abusers; female offenders; and internet offenders (9). In this paper, the characterization made by Barroso et al. (10) will be used, whereas sex offenders' criminal and deviant sexual behavior can be categorized into three groups: (i) violent sex offenders, perpetrated with the use of strength, threat, violence, authority position and lack of consent (e.g., rapists); (ii) child abusers, who commit sexual offenses against children; and (iii) child abusers with pedophilia (and possibly other paraphilias).

Nevertheless, sex offenders have some common characteristics (11). In general, they are people with a history of neglect or abuse, whether physical, emotional or sexual (12). They often have a lack of social or emotional skills (e.g., impaired impulse control) (13), mental disorders such as depression, anxiety, substance use and abuse (14) or antisocial personality disorder, and intellectual disabilities or neurological deficits (11). Additionally, these individuals tend to have a set of cognitive distortions and beliefs (15) usually false (i.e., rape myth), which may also contribute to their deviant behavior (14). As a result, many sex offenders do not recognize the illegal nature of their behavior, do not admit responsibility for it, or claim that the act was consensual and/or due to the victim's behavior, so the risk of recidivism is high (16). Therefore, it is imperative to carry out an adequate risk assessment, to reduce re-offenses and reintegrate the offenders into society (17).

Sexual offense has also a major impact on the media and on the (re)formulation of criminal policies [e.g., (18)]. As a result, all over the world, the governments of different countries (e.g., European and North American) have been adopting more restrictive measures to control the behavior of this population. In addition to prevention and awareness campaigns (19), treatment programs have also been increasing (11).

The first approaches to treating offenders, implemented in the late 1960s, were mostly psychoanalytic. Although the goal was to help the offender identify and resolve early conflicts or trauma, which were thought to be the cause of the offending behavior, the results obtained were not adequate (20). This led part of the scientific community to argue that the treatment of these individuals would be difficult, or even useless (i.e., nothing works) (21), leading to choosing aversive techniques when it came to dealing with offenders (e.g., punishing the offender using electric shock) (22).

Over the years, several other interventions have been tried with the ultimate goal of reducing sexual violence [e.g., (23)]. In addition to strengthening punitive sanctions, establishing laws, and registering offenders, psychological and pharmacological treatments for offenders have also gained status. Particularly, psychological treatment for sexual offender has been widely studied, and the recidivism of sex offenders is one of the main results when evaluating its effectiveness (5). Pharmacological treatments (such as antidepressant medication therapy and testosterone lowering medications) have been developed using the principle of intervention of the reduction of total and free testosterone in the endocrine system, significantly reducing sexual drive and consequently sexual behavior, including deviant sexual behavior (24). Curiously, the World Federation of Societies of Biological Psychiatry (WFSBP) proposed that pharmacological interventions should always be part of a more comprehensive treatment plan (e.g., CBT), since the combination of pharmacological and behavioral treatment coupled with close legal supervision appears to reduce the risk of repeated offense (5).

With the overall objective of treating sex offenders to prevent individuals from engaging in further sexual victimization, there has been, in this regard, international progress in research and practice in treating sex offenders, although the question of “What Works?” for sexual offenders is still discussed (25, 26). The discussion is complicated by many factors that can impact the empirical findings, for instance, different types of offenses, groups of offenders, comorbidities, content of treatment, quality of implementation, assessment designs, outcome criteria, legal regulations, and institutions national contexts [e.g., (11, 27)].

As a consequence, evaluations of treatment programs for sex offenders have so far produced mixed results on the effectiveness of such interventions, and no comprehensive conclusions can be drawn from any individual study (28). In fact, although numerous studies have been carried out on the effectiveness of treatment for sex offenders in sexual recidivism, some have found positive results (of certain types/modalities of interventions) [e.g., (18)], while others have reached neutral or negative results [e.g., (25, 28)]. In general, many involved small samples and/or low-quality control groups (28). There is a widespread agreement that there is a need for high-quality evaluations of treatment programs for sex offenders [e.g., (18, 25)], and the lack of high-quality evidence is so great that if one were to consider only high-quality published studies, there would be virtually no evidence that treatment programs can reduce sexual recidivism (28).

Some literature reviews have been carried out to gather evidence regarding the impact of adult male sex offenders' psychological treatment in reducing recidivism, some of them over the last decade. Schmucker and Lösel (18) conducted a meta-analysis of relatively well-controlled outcome assessments evaluating the effects of psychosocial treatment for male sex offenders to reduce recidivism, comparing treated sex offender groups with equivalent control groups. Overall, there was a positive and statistically significant effect of treatment on sexual recidivism; CBT programs showed a significant effect and other types of intervention showed weaker or no effects. In addition, they found significant effects for treatment in the community and in forensic hospitals, but there is still not enough evidence to draw conclusions about the effectiveness of treating sex offenders in prisons.

Aiming to measure the effect of treatment on sex offenders, Soldino and Carbonell-Vayá (29) developed a meta-analysis from seventeen studies, containing a total sample of 6,681 sex offenders. The obtained rates of sexual recidivism and overall recidivism of treated offenders demonstrated the ability of psychological treatments to reduce the risk of sexual and general recidivism of sex offenders. However, and according to the authors, the interpretation of such results requires caution, as an independent analisys of the studies with good methodological quality did not show significant treatment effects.

Långström et al. (30) produced a systematic review to assess the effectiveness of current medical and psychological interventions designed to prevent reoffending among known abusers and prevention for individuals at risk of sexually abusing children. They included eight studies, all randomized controlled trials and prospective controlled observational studies (cohort studies, follow-up studies, or case-control studies with prospectively collected data) of adult or adolescent perpetrators or potential perpetrators of child sexual abuse and studies of children with sexual behavior problems. As a result, they found weak evidence for interventions aimed at reducing recidivism in identified child sex offenders and inadequate evidence regarding effectiveness of treatment for children with sexual behavioral problems in the one trial identified.

Dennis et al. (25), updating a previous Cochrane review (with a new protocol), sought to assess the effects of psychological interventions on sex offenders or those at risk of committing a sex offense. For this, they included ten randomized clinical trials involving data from 944 adults, all male, treated in institutions (prison or psychiatric center) or in the community. Their main conclusion was that there was no evidence from any of the studies in favor of active intervention in reducing sexual recidivism.

Therefore, while the effectiveness of treatment for sex offenders remains a topic of scientific and professional debate (18, 25), there is evidence that the most effective psychological interventions follow the Risk-Need-Responsivity (RNR) principles proposed by Andrews and Bonta (31).

The RNR state that the intensity of a treatment should be proportionate to the risk of recidivism, that treatment should address problems related to recidivism, and that treatment should be consistent with the offenders' culture and learning style (32). One recommendation is that the treatment must be adapted according to: (i) the risk level and (ii) the crime-related needs (i.e., criminogenic needs). In addition, factors such as education level, learning ability, motivation for change, level of risk, and the type of crime committed should be addressed during the treatment. For example, higher risk offenders (i.e., more likely to reoffend) should receive more intensive treatment [e.g., (33)]. Finally, the RNR proposed Cognitive Behavioral Therapy (CBT) as the most suitable approach for treating these and other types of offenders (31). The main objective is to reduce the risk, using different techniques/strategies, such as: (i) restructuring cognitive distortions, mainly those related to sexual offense; (ii) promote life skills (e.g., empowerment); (iii) reduce impulsivity; and (iv) decrease deviant sexual fantasies and arousal (7, 11). Promoting insight and coping skills to deal with day-to-day adversities is another concern at CBT (7).

Regarding needs, the RNR suggests that the treatment goals should be driven by the dynamic risk factors, or criminogenic needs. These have been empirically proven to be linked with recidivism risk and are amenable to change. On the contrary, static risk factors are predictive of the recidivism risk, but are not suitable to change as they are historic and related to the life stories of the sex offenders. Several dynamic risk factors have been established by research, but one can reduce them to four domains: sexual arousal factors (e.g., deviant sexual interests, preferences); attitudes tolerant of sexual assault (e.g., frequent thinking about sexual activity; cognitive distortions and attitudes that legitimate abuse); interpersonal deficits (e.g., antisocial preferences; lack of social competences; deficits in intimacy; difficulties with victim empathy); and self- regulation deficits (e.g., difficulties in emotional and anger regulation; hostile and impulsive facets) (20, 34).

### The present study

Given the evidence presented, regardless of the treatment model or the post-release program, prevention of recidivism and the (re)integration of individuals into society seem to be the main goals of interventions, as well as the effective management and supervision of sex offenders (35, 36). However, as noted, current treatment programs have shown divergent and sometimes contradictory findings regarding their effectiveness (18), and in particular, their impact on reducing recidivism.

There are a considerable number of offenders that, even after being treated in prison, reoffend when released. Others might not even be integrated into a treatment program, and social re-entry might be difficult, which increases their risk level for reoffending (37). In addition, the release of a sexual offender is a matter of concern within communities. So, what is offered in terms of treatment in this setting is even more important. Most studies on intervention outcomes with sex offenders analyzed several prison contexts, community, or other institutions (e.g., hospitals). There are no findings that report the recidivism rates only in community settings treatments.

Community-based interventions are defined as an aftercare following custodial treatment and/or release from prison (38). Community-based interventions have as a main goal the protection of past victims and the prevention of future victimization. To this end, community programs should be able to: (a) adequately assess the risk the offender represents for the community; and (b) continually assess the offender's likelihood of committing future offenses (39). Thus, community programs for this population must be the result of collaborative work of treatment, followed by management and supervision, carried out by different agents (e.g., police, psychologists, defense lawyers, judges, social service workers, family members of criminals) (40). This type of intervention must, among several aspects: (a) select and carry out the appropriate treatment for the offender (e.g., psychological, pharmacological), monitoring their level of commitment; (b) assess the offender's place of residence and employment, as well as him/her leisure activities (e.g., if he/she is engaged in inappropriate and high risk behaviors); (c) establish restrictions and obligations that diminish the likelihood of re-offense (e.g., contact with minors or other potential victims), considering the offense committed; and (d) verify the offender's social network, such as friends and family members who are aware of his/her criminal history, in order to support the community supervise plan and identify sex offender's risk factors (40).

Research based in the United States and Canada supports the notion that offenders who are released from prison may benefit from support during re-entry and social reintegration. Specifically, they appear to benefit from assistance that meets their survival-based needs, as well as skills training services that maximize the offender's likelihood of securing employment and financial stability (7). With such community-based support, many of the barriers to successful reintegration faced by sex offenders can be mitigated. However, when released, sex offenders face many risk factors related to the sexual offense (e.g., lack of social skills, internet use, reduced social support, among others). As a result, some community intervention programs are offered to this population, hoping to promote skills that prevent relapse that can be at the same time applied in their lives, which does not happen in prison settings. These facts justify the scientific and social relevance of this systematic literature review.

## Materials and methods

### Research question

This systematic literature review aimed to answer the following research question: does community treatment programs for sex offenders reduce recidivism? We aimed to understand: (i) what psychological intervention approach is used in community settings programs, as well as their duration and content; and (ii) what is its impact on outcomes, primarily on recidivism rates and on the targeted criminogenic needs.

### Target population

This systematic review focuses on individuals, of any age and gender, convicted by sexual crimes and released after serving all or part of the sentence, and have been in psychological treatment in a community setting.

### Eligibility criteria

To select the studies, inclusion and exclusion criteria were followed. Inclusion criteria were: (i) quantitative empirical studies in which (ii) a community intervention program targeted (iii) sex offenders. Studies referring to interventions that targeted other samples such as the relatives of individuals who sexually offended or the professionals working with sex offenders in correctional or community settings were excluded. Although no temporal or geographical restrictions have been applied, the selected studies had to be (iv) written in Portuguese, English, French, or Spanish. In addition, they had to be (v) published in academic and peer-reviewed journals. Exclusion criteria were: (i) theoretical and case studies, narrative reviews, systematic reviews and meta-analyses, books, reports, dissertations or theses, comments, conference abstracts, and (ii) instrument validation studies.

### Search expression

The following expression was used, with the necessary adjustments to each database: TI (intervent^*^ OR strateg^*^ OR “best practices” OR treat^*^ OR therap^*^ OR program^*^ OR manage^*^) AND TI (“sex offend^*^” OR “child sex abus^*^” OR “rapist^*^” OR “adult^*^ offend^*^” OR “sexual abus^*^” OR “pedophilia^*^” OR “you^*^ sex offend^*^”) AND TI (community OR “community-based”).

### Study selection and data extraction

This systematic review was conducted according to the Preferred Reporting Items for Systematic Reviews and Meta-Analyses (PRISMA) guidelines (41).

Studies were selected up to January 2022 in multiple databases, namely EBSCO, PubMed, and Web of Science, according to the previous search syntax. Additionally, a manual search was carried out to identify more articles related to the theme.

The search focused on the titles and abstracts of identified articles and was conducted by two independent reviewers. A senior researcher was involved only to solve discrepancies, reducing the probability of errors in study selection. Cohen's Kappa revealed an almost perfect agreement index between reviewers (*K* =.90, *p* < 0.05) (42). Then, a full-text analysis of eligible articles was performed.

**Figure 1:**
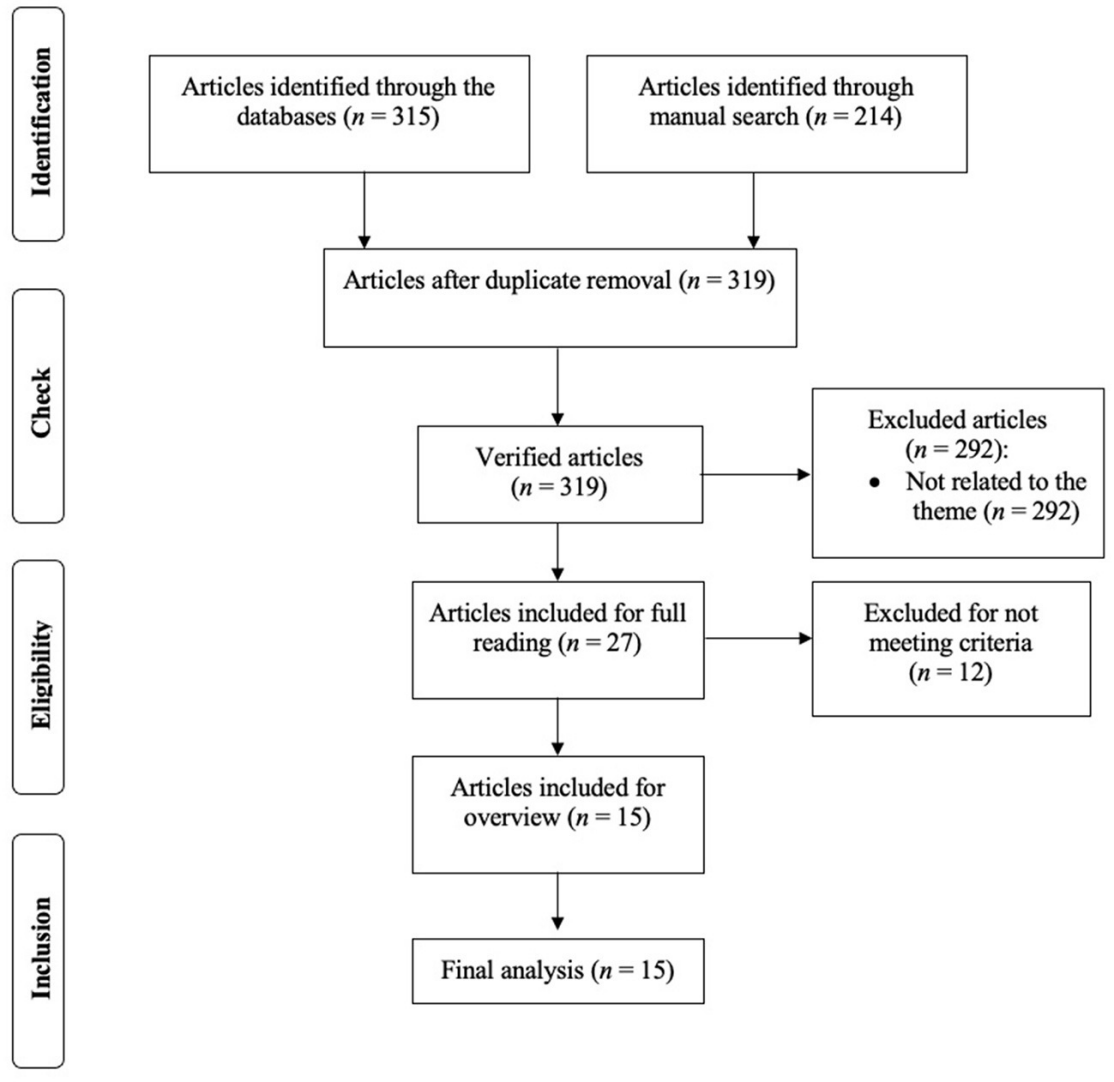
Diagram of flow.

After removing duplicates, 76 articles were identified from EBSCO, 28 articles were identified from Web of Science, 5 articles were identified from PubMed, and 210 articles were identified through manual research. Therefore, a total of 319 studies, published between 1978 and 2022, were identified. From the abstracts and/or titles analysis, 27 articles were retained for full-text analysis. Twelve were then excluded because *n* = 9 were theoretical studies; *n* = 1 was a book; and *n* = 2 were another type of study. At the end, this systematic review comprised 15 articles (Figure 1), from which publication data, objectives, methodological aspects (e.g., age, sample type, and instruments), intervention characteristics, results, and main conclusions were extracted.

### Quality assessment

The Quality Assessment Tool for Quantitative Studies (43) was used to assess the methodological quality of the studies included in this review. The tool assesses: (a) selection bias; (b) study design; (c) confounders; (d) blinding; (e) data collection methods; and (f) dropouts. After each item received a mark ranging between “strong”, “moderate”, and “weak”, quality global rating was defined upon the following criteria: 1. strong = no weak ratings; 2. moderate = one weak rating; 3. weak = two or more weak ratings (Table 1).

**Table 1:** Quality assessment ratings of included studies using the EPHPP quality assessment tool for quantitative studies.

**Study ID**	**Selection** **bias**	**Study** **design**	**Confounders**	**Blinding**	**Data** **collection**	**Withdrawals/** **dropouts**	**Global** **rating**
Bates and Metcalf (44)	2	2	3	1	1	2	**2**
Beech et al. (45)	2	2	2	3	1	2	**2**
Bitton and Abulafia (46)	2	2	1	1	1	2	**1**
Buttell (39)	2	3	1	2	1	3	**3**
Craig et al. (47)	2	2	3	1	1	1	**2**
Craissati et al. (48)	2	3	1	1	1	2	**2**
Hanson et al. (49)	2	1	1	3	1	3	**3**
Harkins et al. (50)	2	2	3	1	1	1	**2**
Lee et al. (51)	2	2	3	3	1	1	**3**
Lussier et al. (52)	2	1	1	2	1	3	**2**
McGrath et al. (53)	2	1	2	3	1	2	**2**
McGrath et al. (54)	2	2	1	2	1	1	**1**
McGrath et al. (55)	2	2	1	3	1	1	**2**
Rose et al. (56)	2	2	1	3	1	2	**2**
Wilson et al. (57)	2	2	1	3	2	3	**3**

**1**: strong; **2**: moderate; **3**: weak.

## Results

Fifteen empirical studies published between 1996 and 2020 were found. Most of them took place in the United Kingdom (UK) (*n* = 6), in the United States of America (*n* = 4), and in Canada (*n* = 3). The remaining studies occurred in Australia (*n* = 1), and Israel (*n* = 1). Two studies used a control group [e.g., (49)], in addition to a treatment group (*n* = 13) [e.g., (45)]. The sample sizes ranged from *N* = 12 (56) to *N* = 777 (50), comprising a total of 3.344 participants. All were men, aged between 15 and 82 years. The mean age ranged from 34.6 (*SD* = 12.5) (55) to 46.1 (*SD* = 11.2) (57), although three studies did not provide this information (44, 50, 51). All studies used forensic samples, that is, individuals previously convicted of committing sexual crimes who, after having served all or part of their sentences, were released, and sent for treatment in the community.

Regarding the objectives, all studies (*n* = 15) explored the effects of the respective intervention and/or treatment programs on recidivism rates, and/or other outcomes (e.g., accepting responsibility, victim empathy, moral reasoning), of different types of sex offenders: (i) violent sex offenders, such as rapists (*n* = 7) [e.g., (52)]; (ii) child abusers (*n* = 5) [e.g., (45)]; and (iii) child abusers with pedophilia (and/or other paraphilias) (*n* = 3) [e.g., (51)].

Regarding therapeutic approaches, 13 studies used Cognitive-Behavioral Therapy (CBT). However, in addition to CBT, some of them used: the relapse prevention model (53, 57), and other approaches (e.g., Applied Behavior Analysis, Psychodynamic therapy) (49). Of the 15 studies, two based their intervention only on the relapse prevention model (52), or on a more recent version of it (50). Motivational techniques (58), for example, were used as an extra [e.g., (56)]. Regarding the length of the treatment and/or follow-up, it ranged from a few months [e.g., (47)], to 12 years (49). The main characteristics of the included studies, interventions, and results are summarized in Tables 2, 3.

**Table 2:** Studies' characteristics.

**Study**	**Country of origin**	**Objectives**	**Sample**		**Instruments**	**CG**
			**Type**	**Age (years)**		
Bates and Metcalf (44)	United Kingdom	To compare psychometric test assessments of a group of men who committed sexual offenses over the internet or by direct contact against a specific victim, both participants in a community-based treatment program for sex offenders (“*Thames Valley Program*” – TVT)	−78 men convicted of sexual offenses against children and adults (e.g., rape). - Divided into two groups: IO (internet offenders): *n* = 39, CO (contact offenders): *n* = 321.	–	−8 psychometric tests, divided into three categories: 1. Offense-specific; 2. Socio-affective; 3. Validity scales.	No
Beech et al. (45)	United Kingdom	To compare the recidivism rates of sex offenders who do or do not respond to treatments made available during the period of probation: (i) “*Community Sex Offender Groupwork Program”* (C-SOGP); (ii) “*Thames Valley Sex Offender Groupwork Program”* (TV-SOGP); and (iii) “*Northumbria Sex Offender Groupwork Program*” (N-SOGP).	−413 men convicted of sexual crimes against children under the age of 14, with or without physical contact, after being released.	*M* = 44.2 (*SD* = 14.2) 18–82.	- CDS; EICS; VEDS; SSES; UCLA ELS; US; PDS; NS; RM2000.	No
			- Divided into three groups according to their treatment needs: low: *n* = 237, intermediate: *n* = 133, high: *n* = 43.			
Bitton and Abulafia (46)	Israel	Explore the efficacy of a community-based treatment provided at a center for adult sex offenders, considering various targeted areas (i.e., accepting responsibility; victim awareness and empathy; emotional regulation, self-monitoring, offense supportive attitudes, intimacy/relationship skills and social skills competences).	−41 men who admitted the practice of sexual offenses, at low to moderate risk levels, with or without a diagnosis of paraphilia (*n* = 21 vs. *n* = 20).	*M* = 37.6 (*SD* = 12.8) 22–70.	- Static-99R; four measurements purposely developed to assess treatment progress.	No
Buttell (39)	United States of América	To investigate the levels of moral reasoning among sex offenders referred by the court for a community-based treatment program.	−72 adult men convicted of various sexual offenses.	*M* = 38	- DIT.	No
			−32% African American and 68% Caucasian.	22–57.		
Craig et al. (47)	United Kingdom	To evaluate a community-based treatment program for sexual offenders with intellectual limitations.	−14 men serving probation orders or prison licenses for being convicted of a contact sexual offense.	*M* = 35.2	- WAIS-III; VABS; BPVS-II;	o
				(*SD* = 14.1) 19–61	- Autism assessment: the diagnostic criteria;	
					- Pre- and post-group intervention: SAK; QACSO; VESA; SOSAS;	
					- RRASOR.	
Craissati et al. (48)	United Kingdom	To explore whether the results of a community sex offender treatment, carried out under the “*Challenge*” project, were maintained in a longer follow-up period.	−273 men convicted of child sexual abuse (*n* = 198) and rape (*n* = 75), at risk in the community.	–	- Static-99.	No
					- Semi-structured clinical interview.	
Hanson et al. (49)	Canada	To evaluate a community treatment program for sex offenders (“*Community Sex Offender Program”* – CSOP), released between 1980 and 1992.	−724 men convicted of sexual offenses or sexually motivated offenses, against adults or children.	EG: *M* = 37.7 (*SD* = 11.2); CG: *M* = 37.0 (*SD* = 11.2)	- Official Canadian Police Records obtained in October 1999.	Yes
			- Divided into two groups: EG: *n* = 403, CG: *n* = 321.			
Harkins et al. (50)	United Kingdom	To assess the effectiveness of the “*Better Lives*” (BL) module included in a recent treatment approach for sex offenders, the “*Good Lives Model*” (GLM), when compared to the standard treatment module focused on relapse prevention (RP).	−777 men integrated into the Northumbrian Sex Offenders Group Work Program (N-SOGP), mostly convicted of sexual crimes against children under 16 (89.1%).	–	- SES; UCLA ELS; IRI; SRI; NS; VES; BACS; ECS; RPQ.	No
			- Divided into two groups: BL: *n* = 76, RP: *n* = 701.		- Semi-structured interview.	
Lee et al. (51)	Australia	Evaluate a community-based treatment program for sex offenders (“*Psychosexual Treatment Program*” – PTP), created by the Community Forensic Psychiatry Service in Victoria in 1989.	−58 men diagnosed with paraphilia, most of them convicted of pedophilia (66%).	*M* = 35.9 (*SD* = 8.6)	- PTPSEF; RS; CQ.	No
Lussier et al. (52)	Canada	Explore the impact of an intensive risk management/oversight program, “*Coordinated High-Risk Offender Management*” (CHROME), on the recidivism of high-risk sex offenders after their release.	−269 male sex offenders divided into four groups: Post-CHROME EG, *n* = 31; Pre-CHROME CG, *n* = 141; Post-CHROME CG, *n* = 82; Pre-CHROME pseudo-control group, *n* = 15)	*M* = 41.2 (*SD* = 13.0)	- Static-99; British Columbia Corrections' computerized data syste.	Yes
				15–80.		
				Post-CHROME EG: *M* = 43.1 (*SD* = 11.0);		
				Pre-CHROME CG, *M* = 40.9 (*SD* = 13.2);		
				Post-CHROME CG, *M* = 41.7 (*SD* = 13.7);		
				Pre-CHROME pseudo-control group, *M* = 37.3 (*SD* = 11.6).		
McGrath et al. (53)	United States of América	Evaluation of a treatment program for sexual offenders (“Vermont Treatment Program for Sexual Aggressors” – VTPSA).	−195 men convicted of sexual or related offense.	*M* = 38.2	- RRASOR; Static-99.	No
McGrath et al. (54)	United States of América	Compare a community-based group treatment program for sex offenders (“*Vermont Treatment Program for Sexual Aggressors*” – VTPSA) with or without polygraph exams.	−208 adult men who had committed sexual offenses, placed under supervision between 1995 and 2001.	*M* = 35.6 (*SD* = 12.8) 18–76.	- RRASOR;	No
			- Divided into two groups: PG (supervision with polygraph exams): *n* = 104; NOPG (supervision without polygraph exams): *n* = 104.	PG: *M* = 35.2 (*SD* = 11.5); NOPG: *M* = 36.0 (*SD* = 14.0).	Static-99; VASOR.	
McGrath et al. (55)	United States of América	Contribute to the consolidation of knowledge on the management of sexual offenders with intellectual disabilities who attended community programs.	−103 men with intellectual disabilities, who received treatment between 1993 and 2004 for sexual offenses against adults or children.	*M* = 34.6 (*SD* = 12.5) 18–70.	- Criminal record checks; RRASOR.	No
Rose et al. (56)	United Kingdom	Develop and evaluate the feasibility of a treatment group for sex offenders with severe intellectual disability in a community setting.	−12 men with intellectual disabilities who committed various sexual crimes (e.g., rape, stalking) against children and adults.	*M* = 39.5 20–65.	- QACSO; NS; SSKAAT-R.	No
Wilson et al. (57)	Canada	To describe a community-based sex offender management protocol, combining parole supervision and treatment focused on relapse prevention to reduce recidivism.	−107 parole sex offenders: *n* = 75 at low risk participated in the relapse prevention maintenance program; *n* = 32 participated in the program for offenders at high risk of recidivism.	- Maintenance program:	- OMS (database); CPIC.	No
				*M* = 45.9 (*SD* = 11.5); High risk offender program: *M* = 46.1 (*SD* = 11.2).		

BACS, Beliefs About Children Scale; BL, Better lives; BPVS- II, British Picture Vocabulary Scale; C-SOGP, Community Sex Offender Groupwork Program; CDS, Cognitive Distortions Scale; CG, Control group; CHROME, Coordinated High-Risk Offender Management; CO, Contact offenders; CQ, Cognitions Questionnaire; CSOP, Community Sex Offender Program; DIT, Defining Issues Test; ECS, Emotional Congruence Scale; EG, Experimental group; EICS, Emotional Identification with Children Scale; GLM, Good Live Models; GSIR, General Information on Recidivism Scale; IO, Internet Offenders; IRI, Interpersonal Reactivity Inventory; N-SOGP, Northumbria Sex Offender Groupwork Program; NOPG, No polygraph group; NS, Nowicki–Strickland Locus of Control; OMS, Offender Management System; PDS, Personal Distress Scale; PG, Polygraph group; PTP, Psychosexual Treatment Program; PTPSEF, Psychosexual Treatment Program Self-Evaluation Form; QACSO, Questionnaire of Attitudes Consistent with Sexual Offending; RM2000, Risk Matrix 2000; RP, Relapse Prevention; RPQ, Relapse Prevention Questionnaire; RRASOR, Rapid Risk Assessment of Sexual Offense Recidivism; RS, Rathus Scale; SAK, Sexual Attitudes and Knowledge Assessment; SD, Standard Deviation; SES, Self-Esteem Scale; SOSAS, Sex Offenses Self-Appraisal Scale; SRI, Social Response Inventory; SSES, Short Self-Esteem Scale; SSKAAT-R, Socio-Sexual Knowledge and Attitudes Assessment–Revised; TV-SOGP, Thames Valley Sex Offender Groupwork Program; TVP, Thames Valley Programme; UCLA ELS, UCLA Emotional Loneliness Scale; US, Underassertiveness Scale; VABS, The Vineland's Adaptive Behavior Scales; VASOR, The Vermont Assessment of Sex Offender Risk; VEDS, Victim Empathy Distortions Scale; VES, Victim Empathy Scale; VESA, Victim Empathy Scale–Adapted; VTPSA, Vermont Treatment Program for Sexual Aggressors; WAIS–III, Wechsler Adult Intelligence Scale-Third Edition.

**Table 3:** Intervention characteristics and results.

**Study**	**Therapeutic approach**	**Intervention description**	**Results and main conclusions**
Bates and Metcalf (44)	CBT	Community treatment program (TVP) that includes: - 2 weeks of intensive group treatment designed to help the offender recognize the deliberate nature of the abuse; - 14 weeks of semi-intensive group treatment focusing on victim's empathy and life skills; - 6 months of treatment focused on relapse prevention. - 160 hours.	- Group of internet offenders with worse results: greater emotional loneliness, less assertiveness, as well as lower scores on external locus of control, sexualized attitudes toward children, emotional congruence, cognitive distortions and empathy for the victim.
Beech et al. (45)	CBT	−3 treatment programs: C-SOGP, TV-SOGP e N-SOGP. - All focused-on victim empathy, life skills, cognitive distortions, and relapse prevention. - 200-hour full version for higher risk/higher treatment-need offenders vs. 100-hour short version for lower risk/lower treatment-need offenders.	−51 participants (12%) reoffended between 2 and 4 years after the end of treatment, 44 of them (86%) in sexual offenses, with or without contact; - No significant differences between groups (needing treatment and responding or not to treatment) in recidivism rates.
Bitton and Abulafia (46)	CBT/RNR	−4 evaluation sessions (2.5/3 h); - Average follow-up of 4.2 years; - Treatment in the various therapeutic groups, individual/family therapy; - Peer group management.	- Most benefited from the treatment as only 2 participants (4.88%), among those who completed the treatment, relapsed. - Significant improvements were observed in the accepting responsibility, victim' awareness and empathy, emotional regulation and in offense supportive attitudes. - Improved understanding of the illegal nature of the acts committed.
Buttell (39)	CBT	- Weekly group community treatment program. It included: (i) confrontation with deviant behaviors and incentives to overcome the strategies used to justify them (e.g., denial, minimization, blame), (ii) cognitive behavioral therapy strategies/techniques (e.g., cognitive restructuring, relapse prevention), and (iii) training in social skills, and focused on empathy.	- No differences in moral reasoning between African-American and Caucasian participants. - Lower levels of moral reasoning when compared to university graduate and high-school students, but no differences compared to elementary school students.
Craig et al. (47)	CBT	Two treatment groups: same treatment facilitators using the same treatment manual, procedures and assessments; - G1: participants living independently in the community (*n* =11); G2: participants living in a probation-approved hostel (*n* = 3); - Group sessions running for two-hours once a week for 14 months; - The core treatment components included: sex education and the law, identifying and reconstructing cognitive distortions, developing victim empathy, relapse prevention skills, the cycle of offending and thoughts related sexual fantasy and masturbation.	- During the follow-up period, none of the men were charged or reconvicted for a new sexual offense; -At the end of the treatment group, 12 group members served the remainder of their probation or license period while under probation supervision without reported incident; - Significant improvements in attitudes toward victims, in perspective taking and victim empathy and a reduction in the use of cognitive distortions and pro-sexual assault attitudes; - Clinically, improvements in “implicit” treatment goals such as listening skills and social responsibility were also evident.
Craissati et al. (48)	CBT	Several modalities: - Structured group treatment, lasting 2 hours per week, from 12 to 15 months, depending on the number of participants. Focused on topics such as disclosure of offense, victim's empathy, cycle of sexual assault and introduction to relapse prevention. - Individual treatment for those who refused the previous treatment or were not able to complete it (e.g., for denying the offense or having a disturbing behavior).	- Reported only data referring to child sexual abusers. - Although not significant, participants who completed treatment were less likely to relapse regarding to sexual and violent crimes (7.11%); - Recidivism rate of 12% for sexual crimes and 14% for violent crimes.
		- Group treatment focused on relapse prevention, lasting 3 days, for those who have completed more extensive treatment elsewhere.	
Hanson et al. (49)	Several (e.g., CBT, ABA, PDT)	The treatment provided (CSOP) varied considering the geographic area: - Program A (*n* = 299, metropolitan area and periphery): group sessions focused on skills training combined with individual sessions focused on problem solving; - Program B (*n* = 45, from the northern region): individual sessions of ABA and behavioral therapy; - Program C (*n* = 41, from small towns or rural areas): group and individual CBT; - Program D (*n* = 15, from urban areas): individual PDT (*n* = 9), or CBT (*n* = 6).	- Similar recidivism rates and no significant differences between participants who received the program and those who did not, after 12 years of follow-up: sexual (21.1% vs 21.8%), violent (42.9% vs 44.5%) and general recidivism (56.6% vs 60.4%).
Harkins et al. (50)	RP/GLM	The treatment consisted of two modules: - CORE module, only for offenders with medium and high risk of deviance, composed of 4 sections focused on content related to the offense, victim' empathy, problem solving, and cognitive distortions. - RP (relapse prevention) or BL module (3 sections, 12 sessions, with motivational techniques, psychoeducation, social and emotional skills training, monitoring and restructuring of unhealthy sexual thoughts and attitudes, etc.) for offenders at low risk of deviance.	- No differences were found between modules regarding attrition rates, or treatment effects on various measures (e.g., recidivism); - Participants from the BL module were more positive about the future and reported having a better understanding of how to avoid future offenses.
Lee et al. (51)	CBT	- Community treatment program (PTP) consisting of 35 weekly group sessions, which took place over 9 months. It included 5 modules: (1) decrease in deviant excitation; (2) social skills and assertiveness training; (3) cognitive restructuring and victim' empathy; (4) relapse prevention; and (5) sex education.	- Positive effects maintained 12 months after program completion; - At the end of the 1-year follow-up only 3 participants reoffended (8.11%); - Improvements in knowledge about sex, expression and communication with adults, accountability, acceptance and problem management, assertiveness, and reduction of cognitive distortions.
Lussier et al. (52)	RP	- Based on the relapse prevention model, the program (CHROME) sought to help the offender to understand his cycle of offenses and to develop adequate coping skills to break this cycle, supporting him with community supervision.	- High relapse rate (35.5%) for participants in the Post-CHROME experimental group, although lower than in the Pre-CHROME pseudo-control group (73.3%). - Group differences only in non-sexual and non-violent recidivism. - Eight sexual offenses (3%) recidivism during the study.
McGrath et al. (53)	CBT RP	- Cognitive-behavioral and relapse prevention program (VTPSA), with further community care and supervision.	- Of those who completed, only 3 (5.4%) committed a new sex offense during the follow-up period. - Participants who completed treatment, when compared to those who did not complete it or who refused it, showed significant differences in sexual recidivism rates (5,4% vs 30.6% vs 30.0%) and in the number of violent crimes.
McGrath et al. (54)	CBT	- Community group treatment, cognitive-behavioral, with correctional supervision (VTPSA), and with or without polygraph exams.	−5-year overall sexual recidivism rate was 6.3%. - Participants who received treatment with polygraph tests had lower rates of recidivism for violent, non-sexual crimes compared to the other group (2.9% vs. 11.5%). No differences for crime of sexual or violent offense.
McGrath et al. (55)	CBT	- Group-administered treatment, based on cognitive-behavioral therapy, included training in social skills, life skills, community participation and sexual risk management.	- During the follow-up period, 11 participants (10.7%) reoffended, mostly in non-contact crimes and with victims known to the offender.
Rose et al. (56)	CBT	−40 sessions per week, lasting two hours. - Focused on sex education and relationships, anger management, cognitive distortions and restructuring, victim empathy, relapse prevention, among others. - Use of motivational techniques.	- Only one (8.3%) of the participants relapsed, 18 months after completing the program; - Significant changes in attitudes and beliefs related to sexual offense; - Significant reductions in attitudes leading to offense.
Wilson et al. (57)	RP CBT	Two programs depending on the level of risk of recidivism: - Maintenance Program: This program, individual, in groups, or combined, according to the offender's needs, was intended for those who have already integrated some treatment and recognized the practice of their crimes. Focused on relapse prevention and risk in the community, with the promotion of understanding of the offense(s) and its impact on victims and the wider community. - High Risk Offender Program: For high-risk offenders. Based on CBT, the group program involves 4 general themes: “Feelings”, “Fantasy”, “Future Planning”, and “Moving Forward”.	- Recidivism rates for the entire sample: general (21%); violent (10.3%); and sexual (3.7%); - High risk group with higher incidence of paraphilias; - Low-risk group with lower risk of general recidivism.

ABA, Applied Behavior Analysis; BL, Better lives; CBT, Cognitive Behavioral Therapy; CHROME, Coordinated High-Risk Offender Management; C-SOGP, Community Sex Offender Groupwork Program; CSOP, Community Sex Offender Program; GLM, Good Lives Model; N-SOGP, Northumbria Sex Offender Groupwork Program; PDT, Psychodynamic therapy; PTP, Psychosexual Treatment Program; RNR, Risk-Need-Responsivity theoretical model; RP, Relapse prevention; TVP, Thames Valley Program; TV-SOGP, Thames Valley Sex Offender Groupwork Program; VTPSA, Vermont Treatment Program for Sexual Aggressors.

### Violent sex offenders

Three studies, carried out in Canada, sought to assess the impact of the intervention programs in reducing recidivism rates among men convicted of several sexual crimes but released after serving part of the prison sentence. Hanson et al. (49) aimed to assess the *Community Sex Offender Program* (CSOP) in a sample of 724 men released between 1980 and 1992 onto community supervision in the Pacific Region of Correctional Service of Canada. To this end, they compared police records on recidivism information of a group of 403 men who were released after receiving the program (treatment group [TG]), with a group of 321 men who did not receive it because they were released before the program was implemented (control/comparison group [CG]). The program was delivered in four different therapeutic models according to geographic region. After a 12-year follow-up period, no between-group differences were observed (*p* > 0.05) in the rates of sexual (21.1 vs. 21.8%), violent (42.9 vs. 44.5%), or general recidivism (56.6 vs. 60.4%).

In turn, Bates and Metcalf (44) aimed to compare psychometric test assessments of men convicted of internet sex offenses (*n* = 39 internet offenders) with men convicted of contact offenses against a specific victim (*n* = 39 contact offenders), whether child or adults. Both offenders were participants of a community treatment program for sex offenders, named *Thames Valley Program* (DVT), which included content focused on helping the offender recognize the deliberate nature of the abuse, the victim's empathy, life skills, and in the relapse prevention. Regarding results, the internet offenders had higher rates of emotional loneliness, lower assertiveness, as well as lower scores in external locus of control, on sexualized attitudes toward children, emotional congruence, and empathy distortions regarding victims of child abuse.

Furthermore, Lussier et al. (52) aimed to explore the impact of *Coordinated High-Risk Offender Management* (CHROME), an intensive supervision program for sex offenders. The CHROME, based on the relapse prevention model, aims to help the participants' understanding of their cycle of offending and developing appropriate coping skills to break this cycle. Two hundred and sixty-nine men were selected and divided into four groups: (i) Post-CHROME experimental group (EG; *n* = 31), who received CHROME services; (ii) Post-CHROME control group (CG; *n* = 82), who did not received CHROME program because their residence was outside the catchment area; (iii) Pre-CHROME control group (PCCG; *n* = 141), who were supervised by probation officers for the duration of their order, but never received CHROME services; (iv) Pre-CHROME pseudo-control group (PCG; *n* = 15), who received services from the CHROME services at some point, but they did not benefit from them at the very beginning of their order. Even though several reoffenses were recorded, with significant differences between groups (35.5% in the EG to 73.3% in the PGC), only a total of eight recidivism in sexual crimes were observed, with a special incidence in the CG (*n* = 4). The results further suggested that, regardless of the type of supervision provided, factors such as age, education, risk level, and the offender's legal status would be predictors of recidivism (*p* < 0.05).

With a similar aim, but developed in the USA, McGrath et al. (53) sought to evaluate the *Vermont Treatment Program for Sexual Aggressors* (VTPSA), a cognitive-behavioral and relapse-prevention treatment for sex offenders. To this end, they examined the recidivism rates of 195 men (*M*_*age*_ = 38.2 years; *SD* = 10.5; *range* = 20–73) who, after being released, were referred for treatment. After a follow-up period of almost 6 years, a significant reduction (*p* < .05) of recidivism was observed among those men who completed the treatment (*n* = 56), when compared with those who dropped out (*n* = 49) or refused treatment (*n* = 90). Thus, and regarding sexual crimes only three people re-offended among those who finished, 15 among those who dropped out, and 45 among those who refused being treated.

Also in the USA, Buttell (39) conducted a study sought to investigate the levels of moral reasoning in sex offenders sent by the Courts for community treatment. It was used a sample of 77 individuals (*M*_*age*_= 38 years; *range* = 22-57), convicted of various sexual crimes. Of these, five were omitted from the analyses due to evaluation inconsistencies, so the results were limited to 72 participants. The results obtained indicated that African Americans and Caucasians had comparable levels of moral reasoning (*p* = 0.85). When compared with the average levels of moral reasoning in the general population, participants had significantly lower levels of moral reasoning than university graduate, and high school students (*p* < 0.001). However, when compared with elementary school students, no significant differences were found (*p* = 0.43). Additionally, they sought to understand the impact of age, level of education, months of treatment, or the number of detentions on levels of moral reasoning. However, no significant differences were found (*p* > 0.05).

In addition, in a study from the United Kingdom, Rose et al. (56), developed and sought to assess the feasibility of a community-based treatment group program for 12 male sex offenders (*M*_age_ = 39.5 years; *range* = 20–65) with intellectual disabilities. Based on cognitive-behavioral approaches, the program included content about sex education and relationships, anger management, cognitive distortions and restructuring, victim empathy, or relapse prevention. Nevertheless, given the cognitive limitations of the individuals, it was essential to work with them in their own homes, as well as to use motivational techniques (58) to promote their participation in the program. In the end, participants showed a reduction in attitudes conducive to and consistent with the offense, an increase in knowledge of sexual issues, and a more external locus of control. In the follow-up period (i.e., 18 months after completing the program), only one of the 12 men offended again.

Lastly, also in the UK, Craig et al. (47) conducted a study to evaluate a 14-month community-based treatment program for sexual offenders with intellectual limitations. For this purpose, they used a total of 14 male convicted sex offenders (*M*_age_ = 35.2 years; *range* = 19–61) serving probation orders or prison licenses. The intervention was based on cognitive-behavioral therapy and comprised of five main components: sex education; cognitive distortions; offending cycle; victim empathy; and relapse prevention. All participants completed psychometric measures specifically designed for people with intellectual limitations before and immediately after completing the treatment program. The results indicated significant improvements in the enhanced insight into the effects of sexual abuse on victims, the perspective taking skills in relation to the offenders' own victims, listening skills and in social responsibility. A reduction in the use of cognitive distortions and pro-sexual assault attitudes was also noted. Of the study sample, 42.8 % (*n* = 6) men were followed up for ~12-months while the rest were followed up to 6 months. In those follow-up periods, none of the group members were reconvicted or recalled for committing a new sexual offense.

### Child abusers

Three UK studies address this sex offender typology. In the first, Beech et al. (45) sought to compare the recidivism rates of those who respond to treatment, with the rates of those who do not respond to the treatments (i.e., available intervention programs), used in different probations areas from the UK: (i) the Community Sex Offender Groupwork Program (C-SOGP); (ii) the Thames Valley Sex Offender Groupwork Program (TV-SOGP); and (iii) Northumbria Sex Offender Groupwork Program (N-SOGP). Additionally, recidivism rates were compared with the respective treatment needs (i.e., low, medium, and high), to verify if the adopted approach was adequate. To this end, they used a sample of 413 individuals (*M*_*age*_ = 44.2 years; *SD* = 14.2) convicted of sex offenses against children (i.e., <14 years), sent by Judicial Authorities for treatment, after their release. Participants were divided into three groups according to the treatment needs, identified in the initial evaluation: (i) low (*n* = 237; 58%); (ii) intermediate (*n* = 133; 32%); (iii) high (*n* = 43; 10%). Regarding the treatment intensity, 212 (90%) of the offenders of low need, 97 (73%) of the offenders of medium need, and 22 (51%) of the offenders of high need, received a shorter dose of treatment. The results indicate that only 51 participants (12% of the total sample) relapsed up to 48 months after the end of treatments (*M* = 30 months; *range* = 24–48), 44 of whom in sexual offenses, with or without contact (i.e., violation of the registration requirements of sex offenders [*n* = 18; 41%], rape [*n* = 5; 11%], downloading indecent images of children [*n* = 3; 7%]), corresponding to 86% of the total number that reoffend. Regarding the recidivism rates, due to treatment needs, or between those who responded or not to the treatments, no significant differences were found (*p* > 0.05), although there was a 40% reduction in the recidivism of those who responded to treatment, compared to the sample considered not to respond to treatment.

In the second, carried out by Craissati et al. (48) sought to find out: (i) whether the results obtained in consultation within the scope of the Challenge Project would be the longest follow-up periods; and (ii) whether the previous results maintained their consistency. To this end, they used the records of 273 sex offenders (child molesters [*n* = 198] and rapists [n = 75]), most of whom (*n* = 145; 53%), at the time, had not received any treatment. Compliance and attendance were two of the variables under study. Thus, 56% of participants adhered to treatment and 59% attended all sessions. Experience of any kind of early trauma (e.g., rape), previous general convictions, and being younger were variables identified as being associated with non-adherence to treatments, so only data concerning child sexual abusers were reported (*n* = 94; *p* < 0.01). Non-compliance was positively associated with later involvement in any type of crime (sexual or violent) and subsequent re-conviction (*p* < 0.05). In contrast, the type of treatment provided was not related (*p* > 0.05). Thus, those who completed treatments were less likely to relapse, although not significantly (*p* > 0.05). They showed a significantly lower propensity (*p* < 0.05) for sexual or violent recidivism. In sum, the results indicated that 12% of the participants relapsed in sexual crimes, and 14% relapsed in violent crimes. In addition, 50% of them practiced some type of formally reprehensible act.

Third, Harkins et al. (50) aimed to compare two sex offender treatment approaches, a standard Relapse Prevention module (RP) with a recent module, the *Better Lives* (BL), derived from the *Good Lives Model* (GLM) rehabilitation theory (59). Both were offered as a second module of the *Northumbria Sex Offender Groupwork Program* (N-SOGP). The first module—the Core module, comprising offense-specific content (e.g., victim empathy, problem solving, and cognitive distortions)—was only offered to those offenders with medium to high-risk level. The risk level was previously assessed *via* the Risk Matrix 2000 (60). Low-risk offenders only attended the second component, either BL or RP module. Hereupon, of 777 men, mostly child abusers (*n* = 692; 89.1%), 76 received the BL module and 701 received the RP module. The aim of RP or BL was to teach the offender alternative ways of behaving and skills to employ when he/she feel vulnerable or likely to reoffend in the future. Although the RP module concentrates on the standard avoidance goals approach, the BL module focused more on approach goals. No significant differences (*p* < 0.05) were observed between BL and RP regarding attrition rates, or in other areas under assessment (e.g., socio-emotional functioning, recidivism). Both were equally effective at retaining participants and achieving change on areas targeted within treatment. Nevertheless, qualitative evaluation data (through semi structured interviews) indicated that BL participants felt more secure about the future and seemed to better understand the strategies to adopt to avoid future offenses.

Two more studies were found, from the USA. McGrath et al. (55) sought to contribute to consolidating knowledge related to the community management of sex offenders with intellectual disabilities. To this end, it was examined the criminal records of 103 men with intellectual disabilities (*M*_age_ = 34.6 years; *SD* = 12.5; *range* = 18–70), who received treatment between 1993 and 2004 because of committing a sexually abusive act (59; [57.3%] of which were against children). The treatments were mostly based on skills training and cognitive-behavioral group therapy. Over an average follow-up period of 5.8 years, 10.7% of the sample (*n* = 11) sexually reoffended. Most re-offenses (55%) were noncontact, and most victims (55%) were known to the offender. No significant differences (*p* > 0.05) were observed between repeat and non-repeated offenders regarding the type of sexual assault, characteristics of the crime, services provided and characteristics of the offenders.

Additionally, McGrath et al. (54) aimed to compare a group of male sex offenders who received the *Vermont Treatment Program for Sexual Abusers* (VTPSA), a community cognitive-behavioral group treatment, with correctional supervision, and with periodic polygraph compliance exams (PG group; *n* = 104, *M*_*age*_ = 35.2 years; *SD* = 11.5), with a group who received the same type of treatment and supervision services but no polygraph exams (NOPG group; *n* = 104; *M*_*age*_ = 38 years; *SD* = 14.0). All participants (*n* = 208; *M*_*age*_ = 35.6 years; *SD* = 12.8; *range* = 18–76) were under state community correctional supervision in Vermont from 1995 to 2001. The results found showed that, after a 5-year follow-up period, the overall rate of sexual recidivism for the all sample was 6.3% (*n* = 13). Those who received the treatment along with polygraph exams committed significantly (*p* < 0.05) less non-sexual violent offense, when compared to those who did not receive polygraph exams (2.9 vs. 11.5%). However, no significant between-group differences were found for sexual, sexual or violent, or any criminal offenses (*p* > 0.05).

### Child abusers with pedophilia and/or other paraphilias

Lee et al. (51), in Australia, sought to evaluate a community-based treatment program for sex offenders, called the *Psychosexual Treatment Program* (PTP), created in Victoria by the Community Forensic Psychiatry Service in 1989. Comprising 35 weekly sessions, carried out in group, over 9 months, the PTP was completed by 37 sex offenders, all diagnosed with paraphilia, and most of them convicted of pedophilia. Based on a cognitive-behavioral approach, the PTP included the following components: (i) decreasing deviant arousal; (ii) social and assertiveness skills training; (iii) cognitive restructuring and victim empathy, (iv) relapse-prevention strategies; and (v) sex education. Upon completion of the treatment, participants showed significant improvements (*p* < 0.05) in their ability to communicate and express themselves with adults, more knowledge about sex, more assertiveness, accountability, acceptance, and problem management, as well as a reduction in sexual arousal, deviant fantasies, and cognitive distortions. These results were maintained 1 year after the completion of the PTP, with recidivism of only three exhibitionists (8.1%), and no pedophiles.

Another study, from Israel, conducted by Bitton and Abulafia (46), based on a CBT approach, and on the principles of the Risk-Need-Responsivity (RNR) theoretical model (31) aimed to assess the evolution over time (i.e., beginning, two intermediate, and final evaluations) of several dynamic risk factors: (i) accepting responsibility; (ii) victim awareness and empathy; (iii) emotional regulation; (iv) self-monitoring; (v) offense supportive attitudes; and (vi) intimacy/relationship skills and social skills competence. Forty-one individuals (*M*_*age*_ = 37.6 years; *SD* = 12.8), who admitted to the practice of sexual offenses, at low to moderate risk levels, were included. They were divided in two groups: (i) paraphilic (*n* = 21); and (ii) non-paraphilic (*n* = 20). The observed results indicated the absence of significant differences between the groups (*p* > 0.05), related to the trends of change in the dimensions evaluated, except for social skills, for which the paraphilic group presented significant changes (*p* < 0.001). Thus, regarding the accepting responsibility, victim awareness and empathy, emotional regulation, and offense supportive attitudes, significant improvements were observed (*p* < 0.05). The analysis of criminal records, among those who completed the treatment, revealed that only two relapsed. In sum, accountability, understanding of the illicit nature of acts or personal danger seemed to have increased. The use of defense mechanisms, characteristic of this type of offenders, was reduced. So, it is reasonable to assume that most participants benefited from the intervention.

Finally, Wilson et al. (57), in a study carried out in Canada, aimed to describe a community-based sexual offender management protocol combining parole supervision and relapse prevention treatment to reduce recidivism. One hundred seven offenders released to the Central Ontario District (Toronto) over an 8-year period were selected. Seventy-five offenders who required low to moderate maintenance of institutional treatment gains (*M*_age_ = 45.9 years; *SD* = 11.5) received the Maintenance Program focused on relapse prevention, while 32 offenders with higher risk for re-offending (*M*_age_ = 46.1 years; *SD* = 11.2) received the High-Risk Offender Program, based on a cognitive-behavioral model, focused on four general themes (feelings, fantasy, future planning, and follow though). At a follow-period (3 years and 7 months), overall rates of 21% for general reoffending, 10.3% for violent reoffending, and 3.7% for sexual reoffending were found, although the results do not clearly separate those diagnosed with pedophilia. Specifically, the maintenance program participants had a lower risk of overall recidivism.

## Discussion

The aim of this systematic review was to identify community-based treatment programs, and their approach, for sex offenders released after serving all or part of their sentences. Most studies referred are in line with the recommendations of the Risk-Need-Responsivity (RNR) theoretical model (31), which suggests the CBT for the treatment of this group of individuals.

Regarding recidivism rates, most treatment programs seem to be effective in reducing it [e.g., (48)], regardless of the approach or theoretical model used (e.g., Relapse Prevention) [e.g., (52)], for male sex offenders. The use of the polygraph could be highlighted since its use is common in the countries of origin of these studies to evaluate the veracity of the offenders' statements. In the study of (54), the use of the polygraph was associated to correctional supervision in order to understand if it would make a difference in reducing recidivism. Indeed, the participants who received the polygraph showed reductions in the rates of recidivism in sexual offenses, when compared to participants who did not receive it, but these differences were not statistically significant. According to the authors (54), the reduction is therefore explained for other factors. First, the participants that received polygraph and supervision spent less time in prison when compared to participants that only received supervision, so that they were probably more motivated to (re)integrate into society. In addition, participants who received the polygraph also completed longer periods of treatment and supervision than those who received only supervision. This study does not give scientific evidence that supports the polygraph use in community treatment, as having a clear effect in the reduction of sexual offense recidivism.

So, indeed, the use of polygraph, at least for sexual offense, might not be directly and significantly related with reduced recidivism rates. This is widely corroborated by recent studies. Authors have even shown that the use of polygraph is associated with smaller treatment effects than programs without polygraph use. This might happen because: (i) the risk of recidivism among those who have committed sex crimes in these studies is so low that the research simply lacks the statistical power to detect a size effect; (ii) the success of polygraph use is associated with the skills of the supervisory professionals who implement it, and they have lack of training in this regard; and (iii) the use of polygraph might undermine a trusting relationship between professional and offender, and as research suggests that those with a history of sexual offenses characteristically deny or minimize their responsibility and truly believe in their innocence, the polygraph test may not detect physiological changes associated with lying [e.g., (61)].

Nevertheless, the positive results were found not only in sex offenders in general, but also in offenders with specific characteristics. Craig et al. (47), McGrath et al. (55), and Rose et al. (56) reported significant reductions in recidivism rates in individuals with intellectual disabilities. In fact, Craig et al. (47) and Rose et al. (56) also showed that their participants improved in other outcomes under study. However, the results should be interpreted with care given the small samples sizes (47, 56). Furthermore, Bitton and Abulafia (46), and Wilson et al. (57) also reported significant reductions in recidivism among offenders diagnosed with paraphilias, including pedophilia. Lee et al. (51), on the other hand, concluded that these results were more evident in participants diagnosed with pedophilia. These results suggest that, despite the difficulties of these individuals, they can be treated if a tailored treatment is provided to them (31). All these results are extremely relevant, as they remain consistent regardless of when the study was performed. The results further suggest that even those who drop out of treatments appear to benefit from the intervention. This result is clear in the study by (53), in which participants who dropped out of treatment had recidivism rates close to those who completed the treatment.

Despite this, Hanson et al. (49) found no significant differences in recidivism rates between treated and untreated, which may have contributed to emphasize the opinion of some authors according to which the treatment of offenders would be useless and ineffective (i.e., nothing works) (62). Nevertheless, such result may be explained since the study included a very large and, possibly, heterogeneous sample in terms of levels of risk and degrees of aggressiveness. Additionally, the possible effect of learning by modeling (63), during the group sessions, cannot be ignored. Furthermore, the follow-up period may have been too long. On the one hand, this may have annulled the beneficial effects of treatment. On the other hand, negative life events may have occurred. Both facts may explain the previous results.

In addition to preventing recidivism, the authors were concerned with assessing the impact of the intervention on other outcomes of crucial importance in the offenders' lives. Thus, after treatments, significant improvements were found in cognitive distortions (44), victim awareness and empathy, emotional regulation (46), listening skills (47), assertiveness, locus of control (50), and in offense supportive attitudes (56). This result is supported by Aslan (64), who also observed that community-based treatments are effective in improving offenders' quality of life.

In this follow-up, there is a result that deserves special attention. Bates and Metcalf (44) indicated that offenders with crimes committed *via* the internet benefited less from treatment when compared to offenders with crimes committed by direct contact with the victim (e.g., they showed less empathy for the victim). This result may be explained by the fact that internet offenders tend to commit less serious offenses (e.g., viewing pornography). Therefore, they tend to present lower risk, less insight and, consequently, lower adherence to treatments and motivation for change (31, 58).

Finally, it should be noted that some studies report high rates of dropout or refusal to participate in treatments [e.g., (53)]. This finding may be related to the coercive nature of this type of intervention. As verified, in many studies there is a clear indication that the offenders were referred for intervention by a competent authority and obliged to participate as a condition to maintain their freedom (65). Therefore, the motivation for change will be reduced. This may compromise participants' adherence to treatments, which, as reported by Craissati et al. (48), is negatively related to recidivism.

This can be explained by the fact that the included studies are, in their overwhelming majority, from Anglo-Saxon countries. The Legal and Criminal system of these countries (i.e., Common Law), is based on Jurisprudence rather than the rule of law (66). This tends to focus on punitive and freedom-restrictive measures and not on the rehabilitation and social reintegration of offenders. Furthermore, the sentences applied to those who break the law tend to be too severe (e.g., life imprisonment, death penalty) (67). As a result, offenders released on parole are required to attend treatment programs, so that they are not arrested again (65).

Additionally, and as already mentioned, due to survival needs, offenders have specific characteristics that differentiate them, such as the ability to manipulate or lie. Therefore, many of them may respond and act in ways to deceive and manipulate researchers. Thus, potential effects of social desirability should not be ignored (68), so the results presented should be interpreted with caution.

In short, the observed results suggest once again the positive effects of CBT in the treatment of sex offenders in community settings, after being released from incarceration. However, these effects can still be improved if relapse prevention techniques (69), motivational interviewing (58), or recommendations from the RNR model are used (31).

Nevertheless, 39 found discrepant results. The author concluded that the level of moral reasoning of sex offenders receiving cognitive-behavioral treatment was similar to that found in school-age children. This result is in line with what is suggested by Berger (70), according to which these individuals tend to have a reduced intellectual development, often similar to that found in children. If not positive, this result is relevant as it may help to explain the poorer results of some studies concerning cognitive-behavioral interventions, as normative moral development is usually necessary for effective change.

### Limitations and potentialities

This systematic review has some limitations, such as the fact that the studies included only male sex offenders. Moreover, and due to the specificity of the theme (i.e., community-based interventions), just a few studies have been published.

On the other hand, results were difficult to compare due to the heterogeneity of the analyzed studies (e.g., diversity of sexual offenses, the wide range of the sample sizes, different instruments used).

Furthermore, the fact that “outpatient” and “ambulatory” were not included as terms that may have captured additional community-based interventions, could represent a limitation for this systematic review. However, even though these terms might contribute for broader research, we considered that they were more related to the medical intervention, so we chose not to include them.

Additionally, the evaluation of the quality of the studies also suggested that 4 of the 15 were of poor quality. In short, in order not to make this systematic review unfeasible, we chose to include them, so the results should be interpreted with caution.

Nevertheless, our study has some potentialities. Based in our review, we confirmed that the treatment of sex offenders in community is overall difficult, but possible. Also, our review seems to demonstrate that CBT-based intervention programs can be useful in the treatment of this population, especially in reducing recidivism rates, as suggested by Olver et al. (17).

### Implications for future studies

Most existing studies tend to focus on heterosexual relationships, in which the man is the offender [e.g., (46)]. However, according to Vandiver et al. (71) the perpetration of offenses, including sexual offenses, is not limited to men, since some women also reveal tendencies to sexually abuse children or adults. In addition, sexual offenses have been reported in homosexual relationships, whether with gays or lesbians (72). Therefore, it would be extremely important to carry out more studies on these populations, which would allow a better characterization of the sexual violence phenomenon.

It would also be important to produce more studies in countries other than Anglo-Saxon ones. According to Gude and Papic (73), restorative justice is very common in the Legal System of these countries (i.e., Civil Law). It tends to favor the reintegration and rehabilitation of offenders, detained or released, and to apply short-term custodial sentences. In this way, individuals may be more motivated to participate and be more involved in treatments (58).

If individuals were more motivated to participate, the dropout rate would probably be lower. As we have seen, some of our studies were of poor to moderate quality, and the high dropout rate was one of the main contributing factors (6 out of 15 studies). Therefore, restorative justice, which implies the use of motivational and reward techniques, could contribute to improving the quality of studies/interventions. The fact that some of the studies did not reveal the number of dropouts and associated reasons also contributed to the lack of quality. This can be explained by the fact that some of these studies are old (that is, publication date exceeds 15 years), and therefore, the scientific community might not be as demanding in terms of disclosing or guaranteeing these aspects as it is nowadays.

The lack of quality was also a result of issues related to blinding, confounders and study design. Some of the studies did not mention whether the outcome assessor(s) were aware of the intervention/exposure status of participants and/or whether the participants were aware of the research question(s); whether there were important differences between the groups prior the intervention, or if there were, whether these differences were strictly controlled; and finally, some of the studies were not carried out under controlled clinical conditions and the tool used to assess quality (EPHPP) naturally scored better for studies in which this happens (e.g., RCT's). Thus, it is clear that a number of methodological issues have to be considered in future studies/interventions, namely, experimental or quasi-experimental study designs, with controllable and appropriate conditions, and reliable data collection and analysis procedures must be explained in detail in the body text of the manuscript.

In addition to all that has been mentioned, it would be relevant to compare the results obtained to better understand the differences between them. If carried out, it would still be expected that potential effects of cultural diversity would be evidenced (74).

## Data availability statement

The original contributions presented in the study are included in the article/supplementary material, further inquiries can be directed to the corresponding author.

## Author contributions

SB and AS conceived the study. SB wrote the initial draft and including all tabular material. CO and EA participated in the initial draft, literature search, data extraction, and wrote the discussion section. DM and FA reviewed the manuscript and including the tabular material. AS supervised the study. All authors read and approved the final manuscript.

## Conflict of interest

The authors declare that the research was conducted in the absence of any commercial or financial relationships that could be construed as a potential conflict of interest. The reviewer VA declared a shared affiliation with the authors CO, DM, FA, and AS to the handling editor at the time of review.

## Publisher's note

All claims expressed in this article are solely those of the authors and do not necessarily represent those of their affiliated organizations, or those of the publisher, the editors and the reviewers. Any product that may be evaluated in this article, or claim that may be made by its manufacturer, is not guaranteed or endorsed by the publisher.
